# Interleukin-33 Contributes Toward Loss of Tolerance by Promoting B-Cell-Activating Factor of the Tumor-Necrosis-Factor Family (BAFF)-Dependent Autoantibody Production

**DOI:** 10.3389/fimmu.2018.02871

**Published:** 2018-12-06

**Authors:** William A. Rose, Angela J. Okragly, Ningjie N. Hu, Montanea R. Daniels, Andrea P. Martin, Yi Ting Koh, Kristine Kikly, Robert J. Benschop

**Affiliations:** Immunology Research, Lilly Research Laboratories, Eli Lilly and Company, Indianapolis, IN, United States

**Keywords:** IL-33, BAFF, autoantibodies, immune tolerance, radiation resistant, germinal center, B cell

## Abstract

Breaking tolerance is a key event leading to autoimmunity, but the exact mechanisms responsible for this remain uncertain. Here we show that the alarmin IL-33 is able to drive the generation of autoantibodies through induction of the B cell survival factor BAFF. A temporary, short-term increase in IL-33 results in a primary (IgM) response to self-antigens. This transient DNA-specific autoantibody response was dependent on the induction of BAFF. Notably, radiation resistant cells and not myeloid cells, such as neutrophils or dendritic cells were the major source of BAFF and were critical in driving the autoantibody response. Chronic exposure to IL-33 elicited dramatic increases in BAFF levels and resulted in elevated numbers of B and T follicular helper cells as well as germinal center formation. We also observed class-switching from an IgM to an IgG DNA-specific autoantibody response. Collectively, the results provide novel insights into a potential mechanism for breaking immune-tolerance via IL-33-mediated induction of BAFF.

## Introduction

Autoimmune diseases span over 80 clinically distinct types of disease and affect 3–5% of the worldwide population with most cases occurring in more developed countries (1–4). Autoimmunity is the result of a dysregulation in the host immune system, involving inappropriate recognition and immune activation against self-antigens (3, 5, 6). The etiology of autoimmune diseases has been linked to environmental and genetic factors, but the exact mechanisms responsible for initiation of autoimmune responses are still uncertain (6–12). Current theories propose that transient insults to the immune system results in generation of autoreactive immune cells and the establishment of an autoimmune state, which potentially requires a chronic pro-inflammatory milieu to develop into a full-blown autoimmune disease (3, 5, 6, 13, 14). Several pro-inflammatory cytokines, such as IL-1β, IL-6, IL-17, and TNF-α have been linked to induction and progression of autoimmune diseases (3, 15).

IL-33, an IL-1 family member, is primarily expressed in cells with barrier function, such as fibroblasts, epithelial cells, and endothelial cells (16–19). Full length IL-33 contains a chromatin binding domain that results in nuclear localization (20, 21). IL-33 is released into the extracellular milieu after cellular damage, mechanical injury, or necrosis and cleaved into a mature cytokine form by a variety of proteases (19, 22–24). The receptor for IL-33 is a heterodimeric complex, consisting of ST2 and IL-1RAcP (25, 26). Consistent with its role as an alarmin, binding of IL-33 to its receptor results in the production of numerous pro-inflammatory factors by a variety of cells types (16, 26). IL-33 can induce a transient inflammatory state and can prime the immune system to generate robust antibody responses (10, 24, 27, 28). In recent years, IL-33 has been linked to several different autoimmune diseases (29–31). Genetic dysregulation of the IL-33/ST2 axis significantly increases the predisposition toward development of inflammatory bowel disease, systemic sclerosis, rheumatoid arthritis and systemic lupus erythematosus (30, 32–35). Chronic inflammation is known to occur in the context of autoimmune diseases with cellular destruction leading to the release of pro-inflammatory stimulants and self-antigens which together promote the development of self-reactive responses (10, 29). A defining characteristic of many autoimmune diseases is the detection of autoantibodies to nuclear components like RNA and DNA (5, 6, 10, 14). Autoantibodies can drive pathology in a number of ways (36) making it critically important to maintain immunological tolerance.

Conversion to autoimmune disease requires a break in self-tolerance, mediated by the activation of self-reactive B or T cells that are normally deleted at selection checkpoints (3, 5, 6). One way that autoreactive B cells can overcome tolerance checkpoints is through overexpression of B cell-activating factor of the TNF family (BAFF) (37, 38). BAFF is an important regulator of B cell survival and maturation (39–41). Previous experiments with transgenic mice overexpressing BAFF showed increased survival of self-reactive B cells with a concomitant production of autoantibodies, resulting in autoantibody-mediated pathology (38, 42–46). Additionally, increased levels of BAFF are detected in the serum of patients with autoimmune disease compared with healthy controls (38, 47, 48). Continuously elevated expression of BAFF thus provides one mechanism for breaking self-tolerance through increased survival of autoreactive B cells.

In this study, we investigated a potential role for IL-33 in the generation of autoantibodies in mice. We find that temporary, short-term increase in IL-33 resulted in a primary (IgM) response to self-antigen. This transient DNA-specific autoantibody response was driven by the induction of BAFF. Notably, radiation resistant cells and not myeloid cells were the major source of BAFF and were critical in driving the autoantibody formation. Continued exposure to IL-33, driven by an adeno-associated virus (AAV) vector system, elicited dramatic increases in BAFF, B and T follicular helper (T_FH_) cell numbers, and was sufficient to break tolerance as evidenced by class-switching from an IgM to an IgG autoantibody response. Collectively, the results provide novel insights into a potential mechanism for breaking immune-tolerance via IL-33-mediated induction of BAFF.

## Material and Methods

### Mice

WT C57BL/6 mice were obtained from Taconic. All animals were housed and maintained at Eli Lilly and Company (Indianapolis, IN). All animal experiments were performed in accordance with the research guidelines approved by the Eli Lilly and Company Institutional Animal Care and Use Committee. BAFF KO mice were generated by Lilly and had a phenotype consistent with published BAFF KO mice (40, 49).

### Short-Term IL-33 Injection Mouse Model

Mice were injected i.p. with 500 ng/mouse (100 μL) of mature form recombinant murine IL-33 (AA102–266, Eli Lilly and Company) or an equal volume of PBS (Thermo Scientific) daily for four consecutive days. For anti-BAFF experiments, mice were injected i.p. with 100 μg/mouse of a neutralizing mouse anti-mouse BAFF antibody (manuscript in preparation) or 100 μg/mouse of mouse IgG1 isotype control antibody (Eli Lilly and Company) 1 day prior to starting IL-33 or PBS injections. For anti-granulocyte-colony stimulating factor (G-CSF) and anti-interferon (IFN)γ experiments, mice were injected i.p. with 250 μg/mouse of rat anti-mouse G-CSF antibody (clone #67604; R&D), 250 μg/mouse of rat anti-mouse IFNγ antibody (clone #R4-6A2; ATCC HB-170) or 250 μg/mouse of rat IgG1 isotype control antibody (Eli Lilly and Company) 1 day prior and with the third dose of daily IL-33 or PBS injection. Serum and spleens were collected 24 h after the last IL-33 or PBS injection or at 1, 2, and 3 weeks after the first IL-33 or PBS injection for further analysis.

### Anti-DNA ELISA

Separate 96 well plates (Greiner Bio-one) were coated with calf thymus DNA (R&D) at 10 μg/mL and incubated overnight at 4°C. Plates were washed with D-PBS (Thermo Scientific) then blocked with D-PBS containing 2% bovine serum albumin fraction V (Thermo Scientific) for 1 h at room temperature (RT). After washing with D-PBS containing 0.05% Tween-20 (Sigma), serum samples were serially diluted in D-PBS containing 2% bovine serum albumin fraction V and 5 μg/mL Heteroblock (Omega Biologicals) then added to the plates and incubated for 2 h at RT. After washing with D-PBS containing 0.05% Tween-20, 1:2,500 dilutions of goat anti-mouse H+L chain HRP (Jackson Immunoresearch), goat anti-mouse IgM (μ chain) HRP (Jackson Immunoresearch) or goat anti-mouse IgG (Fcγ) HRP (Jackson Immunoresearch) were added to the plates and incubated for 90 min at RT. Plates were washed with D-PBS containing 0.05% Tween-20 then developed for 5 min with SureBlue Reserve TMB substrate (SeraCare). Reactions were stopped using 1 N HCL (Thermo Scientific) and plates were read at 450λ using a Molecular Devices Spectra Max 340 PC running Soft Max Pro 3.1.2.

### Mouse BAFF ELISA

Separate 96 well plates were coated with mouse anti-mouse BAFF antibody at 1 μg/mL and incubated overnight at 4°C. Plates were washed with PBS then blocked with casein (Thermo Scientific) for 1 h at RT. After washing, serum samples were diluted 1:10 in casein then added to the plates along with standardized dilutions of mouse BAFF (Eli Lilly and Company) and incubated for 2 h at RT. After washing, 1 μg/mL of biotinylated rat anti-mouse BAFF antibody (biotinylated clone #121808; R&D) was added to the plates and incubated for 1 h at RT. Plates were washed and a 1:2,000 dilution of streptavidin HRP (Jackson Immunoresearch) was added to the plates and incubated for 30 min at RT. Plates were washed then developed for 5 min with SureBlue Reserve TMB substrate. Reactions were stopped using 1 N HCL and plates were read at 450λ using a Molecular Devices Spectra Max 340 PC running Soft Max Pro 3.1.2. BAFF concentrations were calculated using a four parameter logistic curve-fit of the standard dilutions.

### Flow Cytometry of Splenocytes

Single cell suspensions of splenocytes were obtained using a 100 μm cell strainer (Thermo Scientific). Red blood cells were removed using Red Blood Cell Lysing Buffer Hybri-Max (Sigma) and by following the manufacturer's instructions. Cells were blocked with CD16/CD32 clone 93 (eBioscience) then stained with the following antibodies from BD, Biolegend or eBiosciences: CD93 BB515 (clone AA4.1), CD43 PE (clone 1B11), IgM PE-Cy7 (clone 11/41), CD5 APC (clone 53-7.3), IgD APC-Cy7 (clone 11-26c.2a), CD19 Pacific Blue (clone 6D5), CD23 BV510 (clone B3B4), CD21 BV605 (clone 7G6). Propidium iodide (Thermo Scientific) labeling excluded dead cells. Fluorescence minus one controls were included to establish gating parameters for CD19^+^ B cells. Flow cytometric data was acquired on a Fortessa running Diva 7.0 software (BD) and analyzed with FlowJo software (FlowJo).

### Mouse Bone Marrow-Derived Dendritic Cell (BMDC) Culture and Stimulation

C57BL/6 mouse bone marrow was isolated and cultured for 7 days to generate dendritic cells as described previously with 10 ng/mL mouse IL-4 and GM-CSF (R&D) also included in the culture media (50). BMDCs were plated (1 × 10^5^ cells/well) in 96 well plates (Thermo Scientific) in RPMI (Thermo Scientific) with 10% heat inactivated fetal bovine serum (Thermo Scientific) and 1% antibiotic/antimycotic (GE Healthcare). PBS (100 μL), IFNγ (R&D) or IL-33 were added at selected concentrations and the plates were incubated at 37°C with 5% CO_2_. Supernatants were collected after 48 h and BAFF levels were quantified via mouse BAFF ELISA.

### Mouse Neutrophil Isolation and Stimulation

Neutrophils were isolated from C57BL/6 mouse bone marrow by using the mouse neutrophil enrichment kit (StemCell) and following the manufacturer's instructions. Neutrophils were plated (1 × 10^5^ cells/well) in 96 well plates in DMEM (Thermo Scientific) with 10% heat inactivated fetal bovine serum and 1% antibiotic/antimycotic. PBS (100 μL), G-CSF (R&D) or IL-33 were added at selected concentrations and the plates were incubated at 37°C with 5% CO_2_. Supernatants were collected after 18 h and BAFF levels were quantified via mouse BAFF ELISA.

### Mouse Bone Marrow Transplantation

Donor WT and BAFF KO bone marrow cells were isolated from femurs and red blood cells were removed using Red Blood Cell Lysing Buffer Hybri-Max. Cells were washed three times in PBS, passed through a 30 μm filter (Thermo Scientific) and counted. Recipient WT and BAFF KO mice were irradiated with 900 rads (Gammacell 40) to achieve complete myeloablation, which was verified by the inability to detect host immune cells by flow cytometry and the fact that mice that did not receive a BM transplant died within 2 weeks post-radiation. Irradiated mice were then injected with 2.5 × 10^6^ cells/mouse via tail vein injection: irradiated WT mice were reconstituted with BAFF KO bone marrow (KO → WT mice) and irradiated BAFF KO mice were reconstituted with WT bone marrow (WT → KO mice). Reconstitution at 6 weeks post-bone marrow transplantation was confirmed in analyzing peripheral blood by flow cytometry to quantify lymphocyte populations with the following antibodies from BD or Biolegend: CD317 PE (clone 129C1), CD19 PerCp-Cy5.5 (clone 1D3), CD11b PE-Cy7 (clone M1/70), CD3 APC (clone 145-2C11), I-A/I-E AF700 (clone M5/114.15.3), CD11c APC-Cy7 (clone HL3), CD4 Pacific Blue (clone RM4-5), Gr-1 BV510 (clone RB6-8C5), CD8 BV605 (clone 53-6.7).

### Chronic IL-33 AAV Overexpression Mouse Model

The chronic IL-33 AAV overexpression mouse model has been described previously (51). Serum and spleens were collected at 1, 2, and 4 weeks after injection for further analysis.

### Statistical Analysis

Statistical analyses were performed using GraphPad Prism software (version 6) and all graphs show mean ± SEM. A *p* < 0.05 was considered significant.

## Results

### IL-33 Induces Formation of Autoantibodies

**Figure 1:**
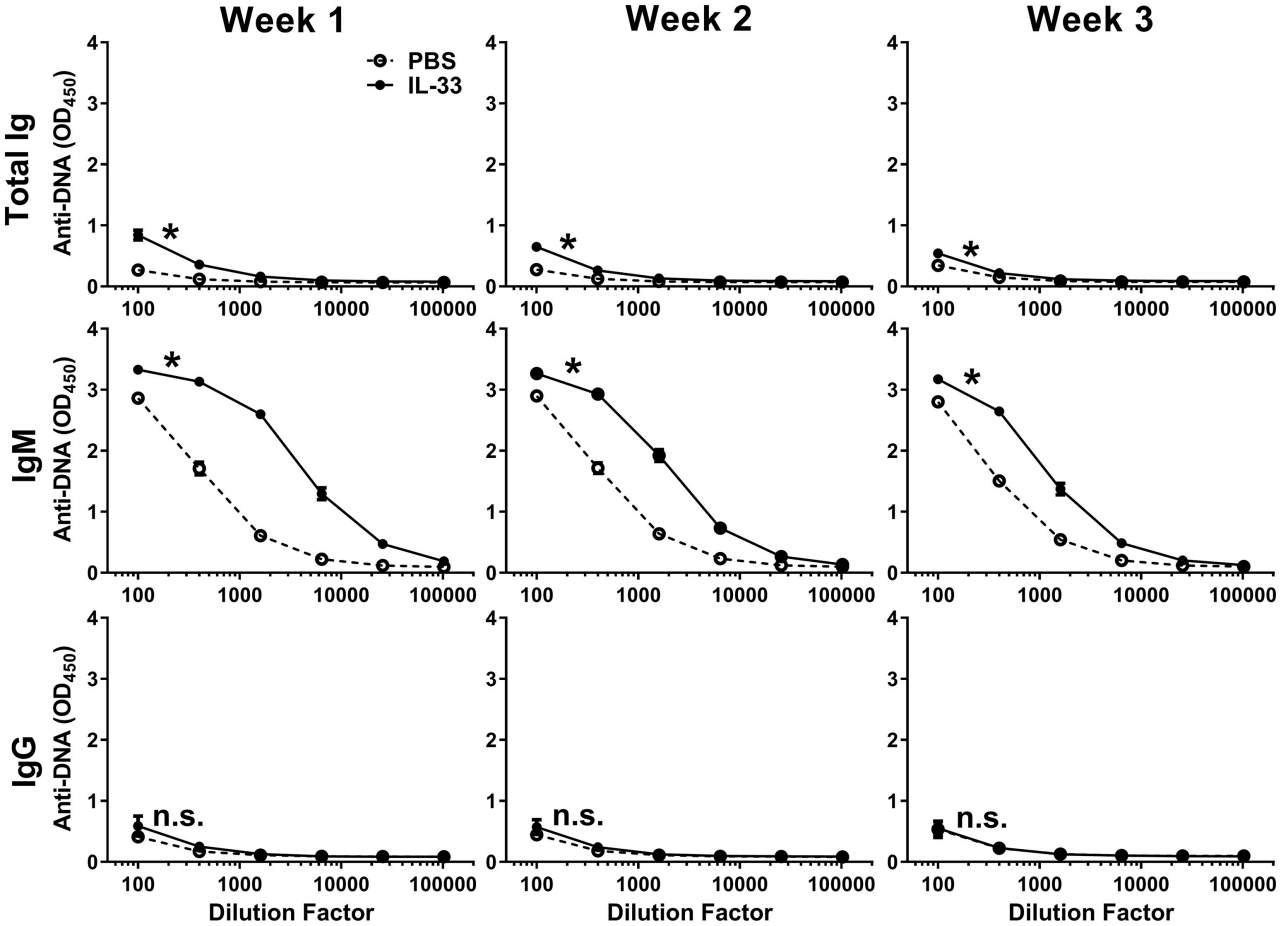
IL-33 induced autoantibody formation. C57BL/6 mice (*n* = 10 mice/group) were injected i.p. with PBS or 500 ng IL-33 daily for four consecutive days. Serum was collected at 1, 2, and 3 weeks after the first IL-33 injection then total Ig, IgM, and IgG anti-DNA titers in PBS (open symbols, dashed line) and IL-33 (closed symbols, solid line) injected mice were determined. Data are representative of two independent experiments. Area under curve (AUC) was determined, comparing PBS to IL-33 injection by two-tailed unpaired *t*-test, *p < 0.05, ns, not significant.

Previous work has shown that IL-33 is released from necrotic cells and that IL-33 can produce adjuvant-like effects (23, 24, 27, 28, 52). Because self-antigens are also released during necrosis (53, 54), we evaluated the possibility that repeated exposure to IL-33 could lead to the formation of autoantibodies. Injection of C57BL/6 mice with 500 ng of IL-33 daily for four consecutive days resulted in an increase in the number of lymphocytes (data not shown) similar to what was reported previously with seven consecutive daily doses of 400 ng IL-33 (16). We quantified anti-DNA titers by measuring total Ig, IgM, and IgG at 1, 2, and 3 weeks after the first IL-33 dose. We observed that IL-33 injections produced significantly higher total Ig titers at week 1, compared with mice injected with PBS (Figure 1). This increase was driven by IgM as no increase in IgG anti-DNA titers were observed (Figure 1). The autoantibody response appeared to be transient as the total Ig and IgM titers decreased at week 2 with a further reduction observed at week 3 (Figure 1). The results show that repeated IL-33 injections induce a transient anti-DNA response.

### BAFF is Induced by IL-33 and Mediates B Cell Expansion and Autoantibody Formation

**Figure 2:**
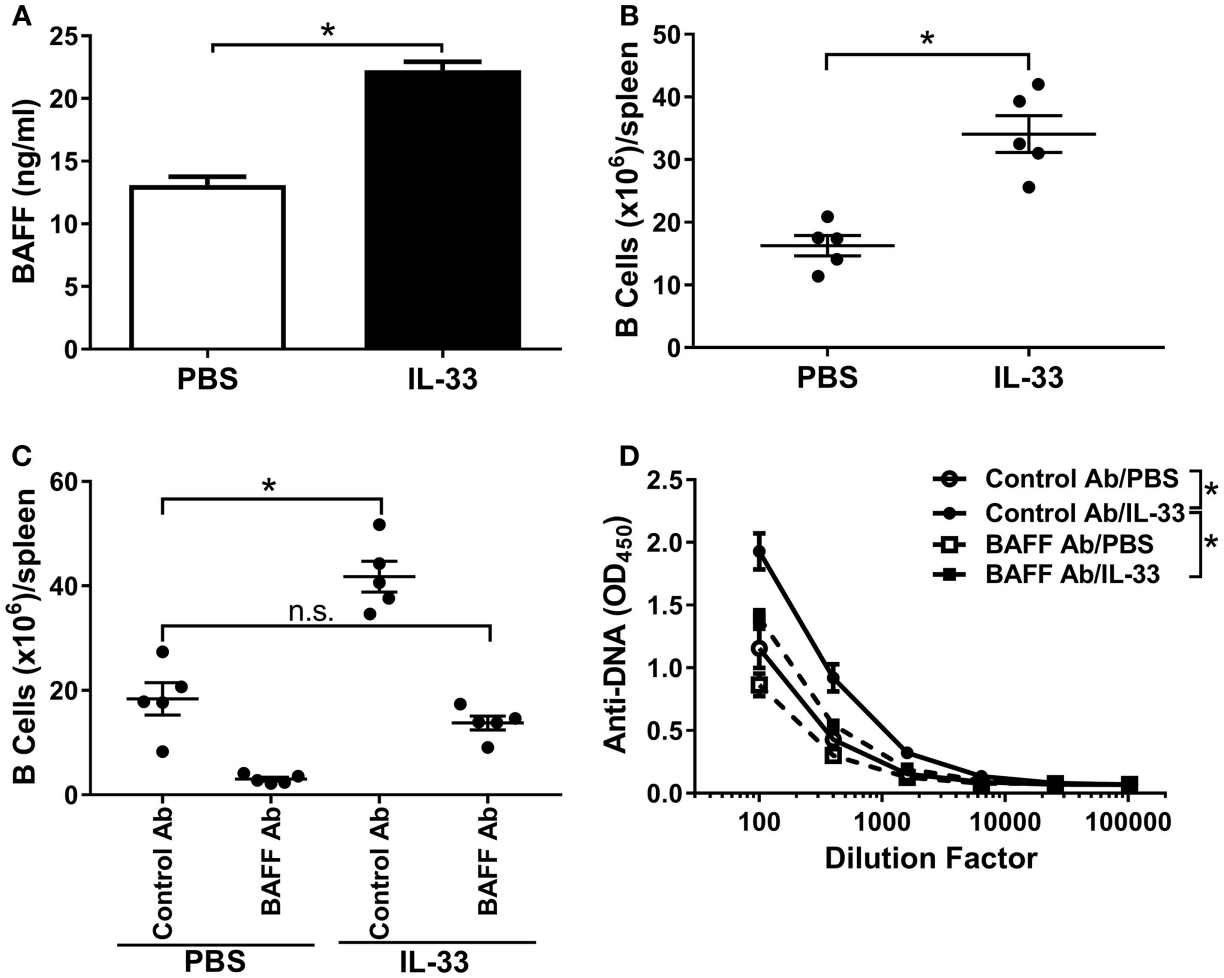
BAFF is necessary for B cell expansion and autoantibody formation. C57BL/6 mice (*n* = 5 mice/group) were injected i.p. with PBS or 500 ng IL-33 daily for four consecutive days. Serum was collected and spleens were harvested 24 h after the last injection then processed for flow cytometry. **(A)** serum BAFF concentrations in PBS (white bar) and IL-33 (black bar) injected mice were determined and **(B)** total (CD19^+^) B cell numbers (*p < 0.05 by two-tailed unpaired *t*-test). **(C)** Total B cell numbers in C57BL/6 mice (*n* = 5 mice/group) treated 1 day prior to four consecutive i.p. injections of PBS or 500 ng IL-33 with mouse IgG1 isotype antibody (Control Ab) or BAFF antibody (BAFF Ab) (*p < 0.05, ns, not significant; One-way ANOVA with Tukey test and four levels of factor). **(D)** Total Ig anti-DNA titers were quantified at different serum dilutions and AUC was determined (*p < 0.05 by one-way ANOVA with Tukey test and four levels of factor). All data are representative of two independent experiments.

Excessive BAFF-mediated survival of B cells can potentially lead to the generation of autoreactive B cells (39, 42). It has been reported that multiple injections of IL-33 in mice increase the B cell population (16, 55). Therefore, we hypothesized a role for BAFF in the observed IL-33-induced autoantibody response. Indeed, serum BAFF levels were significantly increased following four consecutive IL-33 injections compared with PBS injected mice (Figure 2A). A corresponding increase in the number of splenic B cells was also observed (Figure 2B). To confirm that BAFF was directly responsible for the observed increases in B cell numbers and anti-DNA antibody titers, we repeated the experiment in the presence of a neutralizing BAFF antibody. Mice were injected 1 day prior to the start of the IL-33 or PBS injection series with either a BAFF neutralizing antibody or an isotype control antibody (100 μg/mouse). As expected, treatment of PBS-injected mice with a BAFF neutralizing antibody caused a significant decrease in the numbers of B cells in the spleen when compared with control antibody (Figure 2C). Consistent with previous results (Figure 2B) we observed that, in mice that were pre-treated with the control antibody, IL-33 injections induced a significant increase in B cell numbers (Figure 2C). In the presence of the BAFF antibody, injection of IL-33 did not result in elevation of the number of B cells beyond what was observed in PBS-injected mice that received control antibody (Figure 2C). In addition, BAFF neutralization prevented the IL-33-induced increase in anti-DNA titers compared with the control antibody treated mice (Figure 2D). These results indicate that an increase in BAFF is responsible for both the increase in splenic B cell numbers and anti-DNA titers following repeated injections with IL-33.

### Radiation Resistant Cells are the Source of IL-33 Induced BAFF

**Figure 3:**
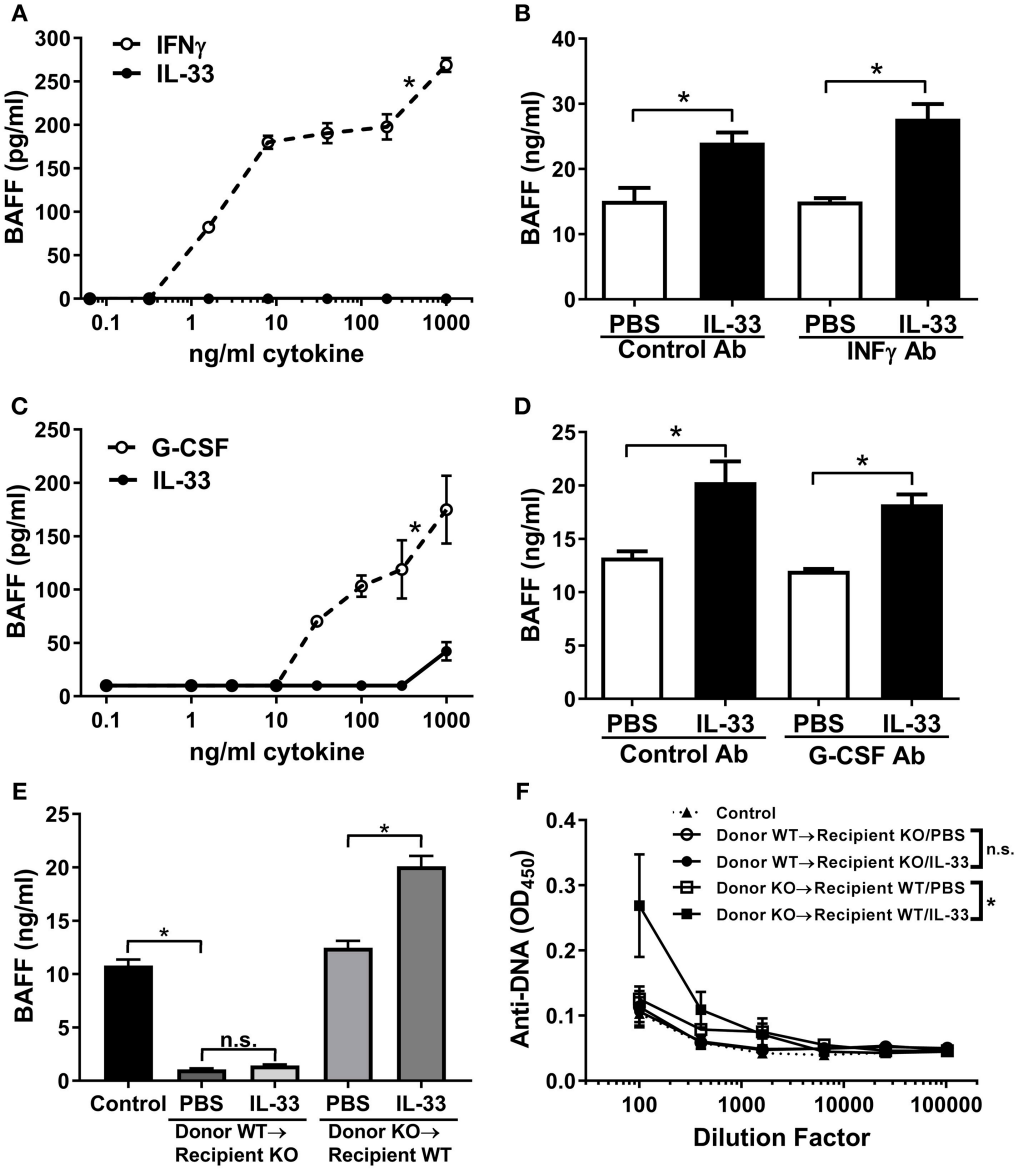
IL-33 induces BAFF production by radiation resistant cells rather than dendritic cells and neutrophils. **(A)** BMDCs from C57BL/6 mice were stimulated with selected concentrations of IFNγ (open symbols, dashed line) or IL-33 (closed symbols, solid line) for 24 h then BAFF concentrations were determined (*p < 0.05 AUC by two-tailed unpaired *t*-test). **(B)** C57BL/6 mice (*n* = 5 mice/group) were pre-treated with rat IgG1 isotype antibody (Control Ab) or IFNγ antibody (IFNγ Ab) injected i.p. 1 day prior to and with the third dose of daily PBS or 500 ng IL-33 injected i.p. for four consecutive days. Serum was collected and BAFF concentrations were determined (*p < 0.05 by one-way ANOVA with Tukey test and four levels of factor). **(C)** Neutrophils isolated from C57BL/6 mice were stimulated with selected concentrations of G-CSF (open symbols, dashed line) or IL-33 (closed symbols, solid line) for 24 h then BAFF concentrations were determined (*p < 0.05 AUC by two-tailed unpaired *t*-test). **(D)** C57BL/6 mice (*n* = 5 mice/group) were pre-treated with rat IgG1 isotype antibody (Control Ab) or G-CSF antibody (G-CSF Ab) injected i.p. 1 day prior and with the third dose of daily PBS or 500 ng IL-33 injected i.p. for four consecutive days. Serum was collected and BAFF concentrations were determined (*p < 0.05 by one-way ANOVA with Tukey test and four levels of factor). **(E)** Six weeks after transplantation of BAFF KO bone marrow into WT and WT bone marrow into BAFF KO mice (*n* = 5 mice/group), mice were injected i.p. with PBS or 500 ng IL-33 daily for four consecutive days. WT mice were used as the control. Serum was collected and BAFF concentrations were determined. No significance (n.s.) donor WT → recipient KO/PBS compared with donor WT → recipient KO/IL-33; *p < 0.05 Control compared with donor WT → recipient KO/PBS and donor KO → recipient WT/PBS compared with donor KO → recipient WT/IL-33 by one-way ANOVA with Tukey test and five levels of factor. **(F)** Total Ig anti-DNA titers were determined at various serum dilutions (comparison of AUC *p < 0.05, ns, not significant by one-way ANOVA with Tukey test five levels of factor). Data are representative of two independent experiments.

It has been shown that dendritic cells and neutrophils are a major cellular source of BAFF (56, 57). Therefore, we investigated whether IL-33 could induce the release of BAFF from dendritic cells and/or neutrophils. While IFNγ stimulated significant release of BAFF from cultured C57BL/6 mouse BMDCs, we observed no production of BAFF upon stimulation with IL-33 (Figure 3A). While IL-33 did not directly act on BMDCs to induce BAFF production *in vitro*, IL-33 could stimulate BAFF production from myeloid cells indirectly via induction of IFNγ from other cell types. To evaluate this possibility, we administered a neutralizing antibody to IFNγ to C57BL/6 mice prior to injection of four consecutive daily doses of IL-33 or PBS. BAFF levels increased equally in mice treated with anti-IFNγ as it did in mice treated with a control antibody upon administration of IL-33 (Figure 3B). A similar set of experiments were used to investigate the role of neutrophils. While treatment with G-CSF resulted in significant release of BAFF from freshly isolated mouse neutrophils, no such increase was observed with IL-33 treatment (Figure 3C). To investigate whether G-CSF was indirectly responsible for the increase in BAFF, mice were treated with a neutralizing G-CSF antibody prior to IL-33 administration. The results showed a similar increase in BAFF levels as observed in mice treated with a control antibody (Figure 3D). In all these experiments, the increase in BAFF was accompanied by a significant increase in B cell numbers (data not shown). Collectively, the *in vitro* and *in vivo* results show that the observed IL-33-induced increase in BAFF does not involve direct stimulation of dendritic cells and neutrophils or indirect stimulation through IFNγ or G-CSF pathways.

Another known source of BAFF is bone marrow stromal cells (58). To elucidate whether stromal cells could be the source of IL-33-induced BAFF, we performed bone marrow transplantation experiments. BAFF KO or WT mice were irradiated and received WT or BAFF KO bone marrow, respectively. Six weeks after bone marrow transplantation all mice had comparable levels of circulating lymphocytes (data not shown), demonstrating adequate reconstitution. Mice in the different groups received either IL-33 or PBS injections and BAFF levels were measured. BAFF KO mice reconstituted with WT bone marrow had significantly lower levels of BAFF compared with control mice and levels did not increase following four consecutive daily IL-33 injections (Figure 3E). In contrast, WT mice reconstituted with BAFF KO bone marrow had normal BAFF levels (compared with control mice) and showed significant increases in BAFF levels following IL-33 administration compared to mice similarly injected with PBS (Figure 3E). These results demonstrate that IL-33 does not stimulate release of BAFF from hematopoietic cells, but rather, from radiation-resistant cells. When anti-DNA titers were measured in the different groups following repeated IL-33 or PBS administration we observed that the total Ig anti-DNA levels were significantly increased only in irradiated WT mice reconstituted with BAFF KO bone marrow (Figure 3F). Notably, BAFF-deficient recipient mice reconstituted with WT bone marrow did not show an increase in anti-DNA titers upon injection with IL-33 (Figure 3F).

### Chronic IL-33 Exposure Induces BAFF, B Cell Expansion and Class-Switching to IgG Autoantibodies

**Figure 4:**
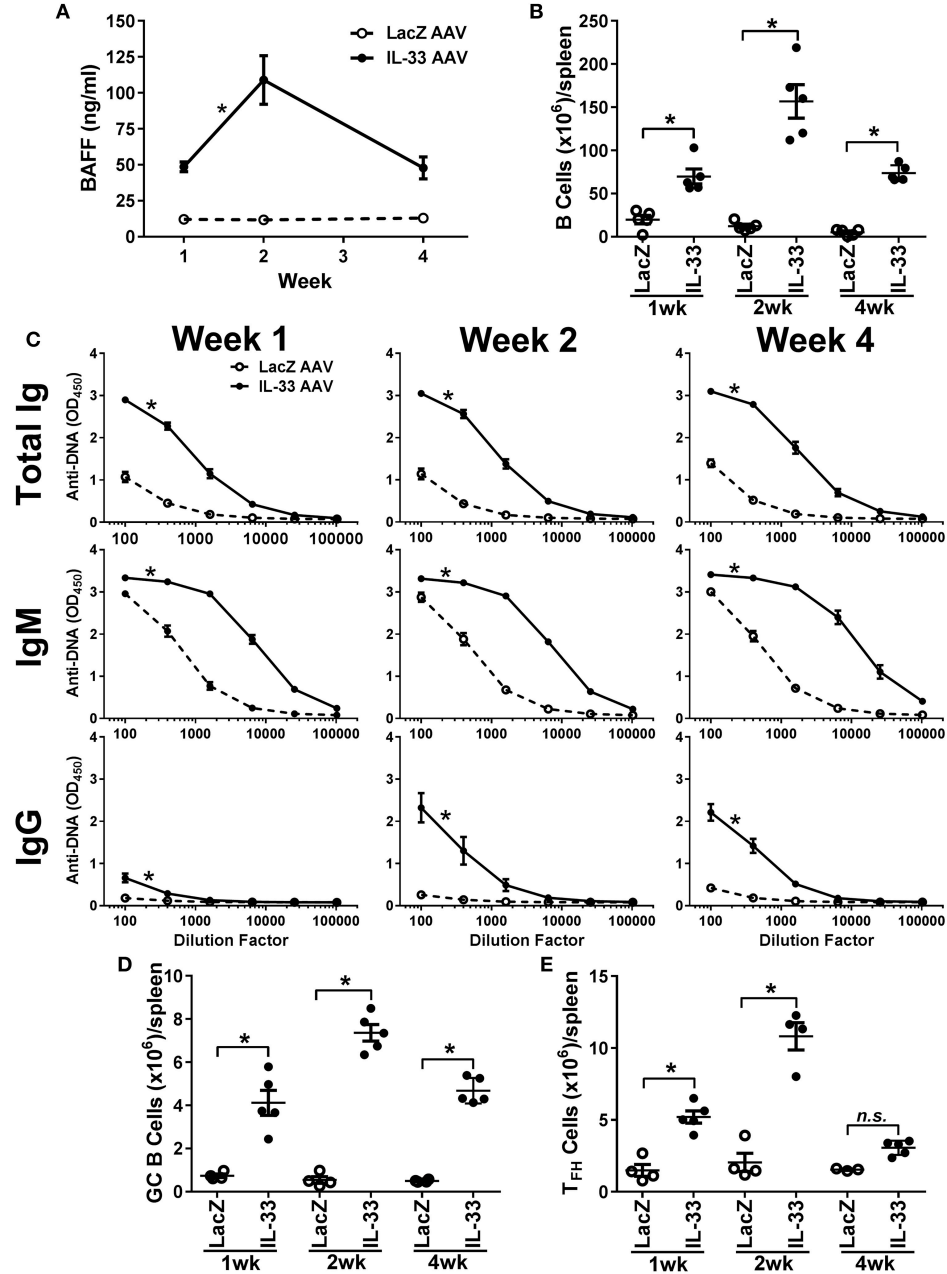
Chronic exposure to IL-33 induces BAFF, B cell expansion, class-switching to IgG anti-DNA antibodies, and increase in GC B cell and T_FH_ cell numbers. C57BL/6 mice (*n* = 5 mice/group/time point) were inoculated with LacZ AAV or IL-33 AAV. Serum was collected and spleens were harvested at 1, 2, and 4 weeks after inoculation. **(A)** BAFF concentrations in LacZ AAV (open symbols, dashed line) and IL-33 AAV (closed symbols, solid line) mice were determined (*p < 0.05 comparing AUC by two-tailed unpaired *t*-test). **(B)** Total B cell counts in the spleen show elevated cell numbers in IL-33 AAV compared with LacZ AAV at all time points (*p < 0.05 by one-way ANOVA with Tukey test and six levels of factor). **(C)** Total Ig, IgM, and IgG anti-DNA titers in LacZ AAV (open symbols, dashed line) and IL-33 AAV (closed symbols, solid line) mice were determined at various serum dilutions. AUC for IL-33 AAV mice was significantly greater where indicated (*p < 0.05 AUC by two-tailed unpaired *t*-test). **(D)** Total CD95^+^GL7^+^ GC B cell counts and **(E)** total CD4^+^CXCR5^+^ T_FH_ cell counts in the spleens were quantified via flow cytometry (*p < 0.05 by one-way ANOVA with Tukey test and six levels of factor). Each symbol represents an individual mouse.

Development of autoimmunity requires a loss of tolerance and class-switching to IgG autoantibodies (59, 60) and, as reported previously, chronic overexpression of BAFF in transgenic mice results in the production of IgG autoantibodies and autoimmunity (42, 45). Since we observed an increase in IgM anti-DNA titers following relatively short exposure to IL-33, we investigated whether prolonged exposure to IL-33 would result in class-switching to IgG. Therefore, we overexpressed IL-33 *in vivo* using an AAV construct and monitored BAFF levels, B cell numbers, and the development of anti-DNA titers over time. IL-33 AAV treated C57BL/6 mice produced significant increased BAFF levels as early as 1 week after infection compared with LacZ AAV treated mice (Figure 4A). BAFF levels continued to increase at week 2. Although BAFF levels started to decline by week 4, they were still significantly elevated compared with LacZ AAV mice (Figure 4A). The increase in BAFF level was accompanied by an increase in the total number of B cells in the spleen of IL-33 AAV mice (Figure 4B). We also observed that chronic exposure to IL-33 induced a robust and prolonged increase in total Ig anti-DNA titers (Figure 4C). IgM specific anti-DNA titers were increased as early as 1 week after IL-33 AAV administration compared to LacZ AAV mice and remained significantly increased throughout the duration of the experiment (Figure 4C). In contrast to the 4-days injection model, chronic exposure to IL-33 induced a significant increase in IgG anti-DNA antibodies after 2 weeks that was maintained through at least 4 weeks (Figure 4C). Similar observations were made in 129 mice showing this is not a strain-specific finding (data not shown). In support of this observed autoantibody class-switching, we observed significant increases in the absolute number of germinal center (GC) B cells (Figure 4D) and T_FH_ cells (Figure 4E) in IL-33 AAV mice over the course the experiment. Collectively, chronic exposure to IL-33 caused an overexpression of BAFF, an increase in the number of B cells, and robust GC formation, resulting in class-switching to IgG autoantibodies.

## Discussion

While genetic and environmental factors are known to be important for the development of autoimmune diseases, the mechanisms behind initiation of autoimmune responses are still uncertain. Here we show that short-term injection of IL-33 in mice elicits a transient IgM autoantibody response that is dependent on IL-33-mediated induction of BAFF. Using bone marrow transplantation, we identified radiation resistant cells as the source of BAFF following administration of IL-33. Chronic exposure to IL-33 results in a correlative increase in BAFF levels and B cell numbers, GC formation, and class-switching to generate a robust anti-DNA response. In rheumatoid arthritis as well as lupus patients, increased serum levels of IL-33 have been shown to correlate with autoantibody levels, including anti-Sjögren's syndrome type B, rheumatoid factor, anti-citrullinated antibodies, among others (29, 61, 62). Other innate mechanisms, including HMGB1 (63) and dsRNA (64), have been shown to induce BAFF production from various cell types. In fact, induction of BAFF via the TLR3 pathway can lead to IgG and IgA class-switching in B cells (65–67). The current data demonstrate that IL-33 is another innate mediator able to provoke the adaptive immune system and break tolerance.

BAFF is an important survival and maturation factor for B cells, and overexpression of BAFF has been linked to autoimmune diseases in mice and humans (38–42, 45, 47, 48) and targeting BAFF and B cells has been a successful therapeutic approach in autoimmune diseases (68). Previous research showed that IL-33 induced expansion of B cell populations (16, 55). Our data expands on this observation to show that IL-33 produces a corresponding increase in serum BAFF that leads to an increase of B cells and an increase in anti-DNA antibodies. We also show that BAFF plays an essential role in this process since BAFF neutralization prevents the expansion of B cells and generation of anti-DNA antibodies. In these experiments, it was noted that B cell numbers in mice injected with IL-33 and a BAFF neutralizing antibody did not fall to the same level as observed in mice injected with PBS and anti-BAFF. Unfortunately, BAFF levels cannot be measured in the presence of a neutralizing BAFF antibody, but the amount of neutralizing BAFF antibody administered was sufficient to capture the amount of BAFF induced by IL-33 in these experiments. Therefore, we hypothesize that this lack of reduction in B cell numbers in this group is due to the induction of other pro-inflammatory factors, such as IL-6 and IL-13 (16, 51), that sustain B cells, rather than an induction in B cell numbers by IL-33.

BAFF is predominately expressed by myeloid cells, specifically neutrophils and dendritic cells (56, 57). It has been shown that administration of G-CSF affects neutrophil biology and that IFNγ induces the maturation of dendritic cells *in vivo* (69–71). Because IL-33 induces both increases in G-CSF and IFNγ (24, 51), it was important to investigate whether the increase in BAFF after IL-33 administration was an indirect result of G-CSF and IFNγ on neutrophils and dendritic cells, respectively. Our data shows that radiation resistant cells are the source of IL-33-induced BAFF, not myeloid cells. While it is possible that our *in vivo* cytokine neutralization strategies for G-CSF and IFNγ were insufficient to definitively rule out that myeloid cells are the source of BAFF, the bone marrow transplantation results clearly demonstrate that radiation resistant cells rather than hematopoietic-derived cells are the source of BAFF that drives autoantibody responses. Of the radiation resistant cell populations, the most likely source of BAFF are bone marrow stromal cells. In addition to being a known producer of BAFF, these cells also express the IL-33 receptor ST2 in both mice and humans (72–74) making it plausible that IL-33 directly stimulates these bone marrow stromal cells. Since IL-33 also induces the expression and release of several other cytokines (26), induction of BAFF could be the result of a combination of direct and indirect stimulation. Regardless, our BAFF neutralization results clearly show that BAFF is necessary and sufficient to drive IL-33-induced generation of anti-DNA antibodies in the short-term exposure model. Furthermore, prolonged exposure to IL-33 induces a break in immune tolerance as shown by a robust GC formation that results in the generation of IgG anti-DNA titers. However, it cannot be ruled out that with chronically elevated levels of IL-33 other factors, besides BAFF, are involved in this process. We have previously shown that IL-6 and TNFα levels increase in this AAV model (51), and these factors could certainly contribute to the observed autoantibody production. We did observe a small and transient, but significant increase in GC and T_FH_ numbers in the 4-days injection model (unpublished observations). This suggests that short transient exposure of IL-33 can lead to activation of nascent self-reactivity via increased BAFF and that chronic stimulation can lead to class-switching.

We and others have shown previously that IL-33 is released from necrotic cells and can exhibit adjuvant-like activity (10, 24, 27, 28). A variety of circumstances like environmental stressors and mechanical damage to cells can results in necrosis and the release of IL-33 along with other nuclear components could activate the local immune system to initiate processes that can result in autoimmunity. In support of this hypothesis, inhibition of the IL-33/ST2 pathway via neutralizing ST2 antibodies or using ST2 KO mice in models of autoimmune disease (e.g., dextran-induced colitis, collagen-induced or autoantibody-induced arthritis) demonstrated reduced severity of disease pathology (75–77). Additionally, inhibition of IL-33 with neutralizing antibodies resulted in reduced pathology in experimental autoimmune encephalomyelitis and MRL/lpr mouse models (78, 79). However, in these reports, the antibodies to IL-33 were rabbit or goat polyclonals that will likely elicit mouse-anti-rabbit or goat responses in these models. Since there were no data on the actual anti-IL-33 antibody exposures during the course of the experiments in these reports, proper interpretation of the results are difficult to make. Furthermore, injections of mice with recombinant IL-33 exacerbated disease pathology in autoantibody-induced and collagen-induced arthritis models, supporting a role for the IL-33/ST2 axis in models of autoimmunity (76, 80). More recently, the functional role of IL-33 has expanded beyond being a potent trigger of inflammation to include a role in tissue homeostasis and repair. IL-33 has been shown to be involved in the stimulation and expansion (maintenance) both regulatory T- (81) and B-cells (82) Given our current and previous (24, 51) findings, it would appear that the context and/or the microenvironment play an important role in determining whether increased levels of IL-33 is associated primarily with regulation or activation. This will require further investigations.

While we demonstrate that IL-33 drives processes leading to loss of immune tolerance, we did not show this leads to autoimmune disease. Another unanswered question is whether IL-33 is required only as an initiator (either acute or intermittent) or whether it is required for prolonged periods of time to drive processes leading to loss of tolerance. However, all of these findings together point to a key role for IL-33 in inducing loss of immune tolerance and current findings indicate that induction of BAFF is a critical event.

## Author Contributions

WR, AO, AM, YK, KK, and RB: study design. WR, AO, NH, MD, AM, and YK: study conduct. WR, AO, AM, YK, KK, and RB: data analysis and interpretation. WR, AO, and RB: wrote the manuscript with all authors providing feedback.

### Conflict of Interest Statement

All authors are or were employed by Eli Lilly and Company. All work was performed at and funded by Eli Lilly and Company.

## Abbreviations

AAV: adeno-associated virus
BAFF: B cell-activating factor of the TNF family
GC: germinal center
G-CSF: granulocyte-colony stimulating factor
T_FH_: T follicular helper
BMDC: bone marrow-derived dendritic cells
